# Using dose aware and unattended data collection modes on the variable and microfocus macromolecular crystallography beamline I04 at Diamond Light Source

**DOI:** 10.1063/4.0000904

**Published:** 2025-10-27

**Authors:** Ralf Flaig, Pierpaolo Romano, David Aragao

**Affiliations:** Diamond Light Source, Didcot, Oxfordshire, United Kingdom

## Abstract

The macromolecular crystallography (MX) beamline I04 [ref 1] at Diamond has evolved over time through various upgrade projects that aimed at increasing scientific capability but at the same time aiming for increased stability so that the best possible data can be obtained by the user. We have implemented an optical concept for beam delivery combining simplicity with stability. Variable focus beam is provided through the combination of a double crystal monochromator (DCM) with a F-switch which houses compound refractive lenses (CRL) that can be brought individually into the beam path, providing maximum flexibility. Both devices were designed inhouse and this combination allows delivery of a very stable beam from the microfocus regime (8 μm x 5 μm (h x v)) to larger beam sizes (up to 110 μm x 100 μm). Beam delivery within 3% RMS of the beamsize is achieved by making use of a dedicated feedback system using X-ray beam position monitors (XBPMs) [ref 2]. The original X-ray source has been replaced by a 17.6 mm period CPMU in June 2022 and has resulted in a significant flux increase over the whole energy range (6-18 keV) thereby generating new scientific opportunities.

Optimal data collection in MX strongly depends on setting up the right data collection parameters which should be defined by the experimental aim or scientific question that is being asked. With high intensity beamlines radiation damage has become the primary limitation and therefore the total exposure of the sample plays a key role. It became soon clear that with the combination of the high variability of the flux profile from the source combined with the large variability of beam sizes, that the concept of exposure per data collection frame is no longer feasible. Therefore, we have implemented the concept of dose aware data collection [ref 3] where the user is given the option to dial a dose per data set (instead of an exposure time per frame) and this dose should of course be compatible with the experimental aim. We use the programme RADDOSE-3D [ref 4, 5] which takes known information from the beamline (energy, flux, beam size) and currently assumes a standard macromolecular crystal which means that the sample only contains lighter elements and combines this information in the dose calculation to produce optimal exposure times per frame and adjustment of transmission if required. Future improvements will consider better information about sample composition and size. In most cases, we aim for the shortest possible exposure time to take advantage of the Eiger2 XE 16M detector capabilities which allows acquisition rates up to 500 Hz. Depending on the experimental aim we usually implement a multi-sweep (and often multi-crystal) approach [ref 6] using different crystal orientations which can be realised with the SmarGon multi-axis goniometer. The dose-based approach is also fully implemented in our unattended data collection (UDC) protocols. Apart from the UDC and remote interactive modes we also strongly encourage in person visits to train users in best practice dose aware data collection and enabling them to make the best choices using the available tools in the data collection software. We constantly aim to streamline the user experience further by addition of new functionality and tools.

## References

[1] https://www.diamond.ac.uk/Instruments/Mx/I04.html

[2] Microfocus and variable beam size at the Diamond Light Source I04 beamline, P. Romano, H. Khosroabadi, D. Aragao, R. Flaig, SRI 2024 conference proceedings, accepted for publication

[3] Dose aware data collection on the variable and microfocus macromolecular crystallography beamline I04 at Diamond Light Source, R. Flaig, P. Romano, D. Aragao, SRI 2024 conference proceedings, accepted for publication

[4] RADDOSE-3D: time- and space-resolved modelling of dose in macromolecular crystallography, O.B. Zeldin, M. Gerstel, E. Garman, J. Appl. Cryst. (2013) 46, 1225–1230 10.1107/S0021889813011461

[5] Estimate your dose: RADDOSE-3D, C.S. Bury, J.C. Brooks-Bartlett, S.P. Walsh, E.F. Garman, Protein Science (2018) 27, 217–228 10.1002/pro.3302 28921782 PMC5734275

[6] How best to use photons, Winter, G. *et al*., Acta Cryst. (2019). D75, 242–261 10.1107/S2059798319003528 PMC6450062 30950396

